# A phenome-wide association study of uterine fibroids reveals a marked burden of comorbidities

**DOI:** 10.1038/s43856-025-00884-w

**Published:** 2025-05-15

**Authors:** Elizabeth A. Jasper, Brian S. Mautz, Jacklyn N. Hellwege, Jacqueline A. Piekos, Sarah H. Jones, Yanfei Zhang, Eric S. Torstenson, Sarah A. Pendergrass, Ming Ta Michael Lee, Todd L. Edwards, Digna R. Velez Edwards

**Affiliations:** 1 Division of Quantitative and Clinical Sciences, Department of Obstetrics and Gynecology, Vanderbilt University Medical Center, Nashville, TN USA; 2 Center for Precision Medicine, Vanderbilt University Medical Center, Nashville, TN USA; 3 Institute for Medicine and Public Health, Vanderbilt University Medical Center, Nashville, TN USA; 4 Vanderbilt Epidemiology Center, Vanderbilt University, Nashville, TN USA; 5 Vanderbilt Genetics Institute, Vanderbilt University, Nashville, TN USA; 6 Population Analytics, Analytics & Insights, Data Sciences, Janssen Research & Development, Spring House, PA USA; 7 Division of Genetic Medicine, Department of Medicine, Vanderbilt University Medical Center, Nashville, TN USA; 8 Genomic Medicine Institute, Geisinger Health Systems, Danville, PA USA; 9 Department of Biomedical Informatics, Vanderbilt University Medical Center, Nashville, TN USA; 10 Division of Epidemiology, Department of Medicine, Vanderbilt University Medical Center, Nashville, TN USA; 11 Genentech, South San Francisco, CA USA; 12 Department of Biomedical and Translational Informatics, Geisinger, Rockville, MD USA

**Keywords:** Urogenital reproductive disorders, Epidemiology

## Abstract

**Background:**

The burden of comorbidities in those with uterine fibroids compared to those without fibroids is understudied. We performed a phenome-wide association study to systematically assess the association between fibroids and other conditions.

**Methods:**

Vanderbilt University Medical Center’s Synthetic Derivative and Geisinger Health System Database, two electronic health record databases, were used for discovery and validation. Non-Hispanic Black and White females were included. Fibroid cases were identified through a previously validated algorithm. Race-stratified and multi-population phenome-wide association analyses, adjusting for age and body mass index, were performed before statistically significant, validated results were meta-analyzed.

**Results:**

There were 52,295 and 26,918 (9022 and 10,232 fibroid cases) females included in discovery and validation analyses. In multi-population meta-analysis, 389 conditions were associated with fibroid risk, with evidence of enrichment of circulatory, dermatologic, genitourinary, musculoskeletal, and sense organ conditions. The strongest associations within and across racial groups included conditions previously associated with fibroids. Numerous novel diagnoses, including cancers in female genital organs, were tied to fibroid status.

**Conclusions:**

Overall, individuals with fibroids have a marked increase in comorbidities compared to those without fibroids. This approach to evaluate the health context of fibroids highlights the potential to understand fibroid etiology through studying the common biology of comorbid diagnoses and through disease networks.

## Plain language summary

Though many females have uterine fibroids, not enough is known about how fibroids affect people’s health compared to those who do not have them. We explored fibroids’ connection to many other health conditions using information from electronic health records and groups of people from different races. Across all groups, we found over 380 conditions were associated with fibroids. Many of these health issues were circulatory, skin, genital and urinary, musculoskeletal, and sense organ conditions. In addition to strong known associations, we also found many diagnoses that have not been associated with fibroids, like cancers in female genitals. Overall, individuals with fibroids have more health conditions compared to those without fibroids. Our approach can help us to understand causes and consequences of fibroids.

## Abstract (web summary)

Jasper and Mautz et al. systematically assess associations between uterine fibroids and hundreds of conditions using electronic health record databases. They identify numerous associations, both known and novel, and find that individuals with fibroids have more comorbidities compared to those without fibroids.

## Introduction

Leiomyomas, also known as uterine fibroids, are benign neoplasms originating in the smooth muscle of the uterus. They are the most common female pelvic tumor developing in up to 80% of females by menopause, and account for up to 34 billion dollars in health care costs in the United States annually^1–4^. Fibroids are the leading indication for hysterectomy^4^. Symptomatic fibroids have a range of reproductive health effect,s including heavy and painful menses, anemia, pelvic pain, and pregnancy complications^5^. However, up to 50% of females remain asymptomatic, complicating research on the etiology of fibroids as asymptomatic cases can be misclassified without pelvic imaging^2^.

Current understanding of the clinical risk factors of fibroids is limited to a small number of candidate risk factors identified primarily from self-reported fibroids or prospective cohorts of imaging-confirmed fibroids. Self-reported Black race is the most well-established risk factor for fibroids, with Black females having 2-fold higher odds of developing fibroids relative to White females^2,4,6^. Black females also develop more numerous and larger fibroids at younger ages^7,8^. Other factors associated with increased fibroid risk include higher body mass index (BMI), family history, a history of hypertension, increasing age, nulliparity, and earlier age at menarche^2,3,9–14^. Smoking has also been shown to be protective in some studies^13,15^.

Phenome-wide association studies (PheWAS) offer a unique way to interrogate comorbid disease and risk relationships on a large scale. PheWAS is a data mining approach that tests for associations between an exposure (such as a genotype or a disease diagnosis) across several available disease phenotypes in a systematic, high-throughput, and reproducible way^16,17^. This method utilizes phecodes, which group relevant International Classification of Diseases codes into clinically meaningful phenotypes and allow researchers to rapidly define phenotypes and query associations. Greater access to long-term information in patient electronic health records (EHRs), combined with PheWAS approaches, will capture relationships not typically collected in traditional cohort studies. PheWAS has been used successfully across several topics, ranging from a study evaluating the relationship between Neanderthal genome and contemporary human phenotypes to studies evaluating the comorbidities associated with systemic lupus erythematosus and leukodystrophies^18–21^. Though traditional PheWAS approaches cannot determine causation since they do not account for temporality, PheWAS provides the opportunity to uncover novel comorbidity associations not possible in typical candidate risk factor studies and allows the assessment of the level of comorbidity burden. Observed associations could then be used to prioritize risk factors for treatment and modification within and across groups.

We used PheWAS to systematically investigate the clinical context of fibroids, to understand broader disease associations and explore the clinical phenome. Our hypothesis was that fibroids status would be associated with known fibroid symptoms, and individuals with fibroids would demonstrate an increased burden of comorbidities. Using two large clinical cohorts, we conducted PheWAS analyses using a previously published and validated phenotyping algorithm that required image confirmation to define fibroid cases and controls^22^. This study was a two-stage design with discovery analyses performed using Vanderbilt University Medical Center’s Synthetic Derivative database, and Geisinger Health Systems EHR database employed for validation of discovery results.

## Methods

### Study cohorts

We utilized Vanderbilt University Medical Center’s (VUMC) Synthetic Derivative (SD) for our discovery analyses. The SD is a de-identified mirror of the VUMC EHRs containing longitudinal data, including demographic and clinical information, for over 3 million subjects who have received care in the VUMC healthcare system^23^. Non-Hispanic Black and White females 18 years or older were eligible for inclusion, with race and ethnicity defined via self-reported or by providers. Cases and controls were identified using our previously published algorithm, which has been shown to have positive and negative predictive values of 96% and 98% respectively^22^. Briefly, cases had at least one International Classification of Diseases, 9th Revision (ICD-9) or current procedure terminology (CPT) code for pelvic imaging and had at least one ICD-9 or CPT code indicating a fibroid diagnosis. Controls had at least two procedural codes for pelvic imaging without a fibroid diagnosis at the time of the last pelvic exam, as well as no history of hysterectomy, myomectomy, or uterine artery embolization.

The Geisinger Health System (GHS) Database was used as a validation cohort, with cases and controls identified by the algorithm described above. GHS is a fully integrated health system serving three million residents of north-central and northeastern Pennsylvania. The database comes from GHS’s physician group practices, which include a network of 1000 physicians across 75 sites, inclusive of 41 community care clinics. As all data was de-identified, this study was deemed non-human subjects research and approved by Vanderbilt University Medical Center Institutional Review Board. All methods were carried out in accordance with relevant guidelines and regulations.

### Statistics and reproducibility

**Fig. 1:**
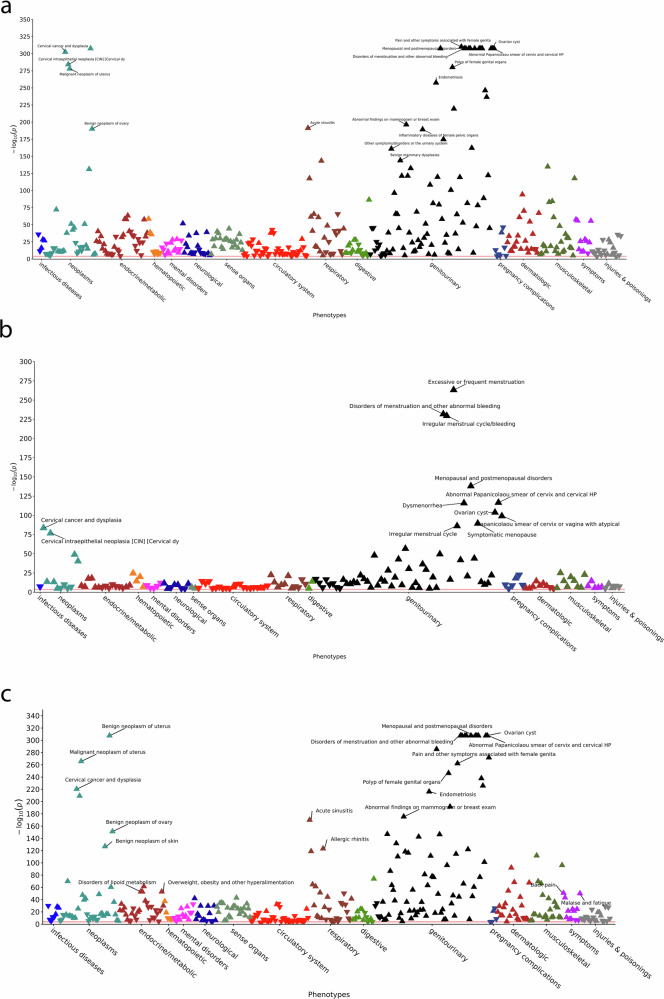
Manhattan plots of PheWAS results from meta-analyzed cohorts. Manhattan plots of phenome-wide association study results for the multi-population [N max = 79,213] (**a**), non-Hispanic Black [N max=11,342] (**b**), and non-Hispanic White [N max=67,871] (**c**) meta-analysis. Panels display validated, statistically significant results that were meta-analyzed across discovery and validation cohorts. Each triangles represents a phenotype. Direction of the triangle’s tip corresponds to increased (up) or decreased (down) odds. The red line indicates statistical significance. Triangles above this line are significantly associated with fibroids.

PheWAS, adjusted for age and BMI, were performed with uterine fibroids as the outcome and each diagnosis, condition, or clinical characteristic (phecode) as an exposure. Analyses were performed using the PheWAS package (v 0.99.5-3) in R version 4.3.2^24^. Discovery PheWAS was first performed in the SD before statistically significant results were validated in GHS cohort (Fig. 1a, Supplementary Fig. 1). A Bonferroni correction based on the number of tests in discovery cohorts was used to determine significance (*p*-value ≤ 2.98 × 10⁻⁵ for non-Hispanic Black individuals, 2.87 × 10⁻⁵ for non-Hispanic White individuals, 2.99 x 10⁻⁵ for multi-population). Phecodes from statistically significant associations in the discovery cohorts were then carried forward through testing in the validation cohorts. Inverse-variance weighted fixed-effects meta-analyses was performed, using METAL software, for associations that were statistically significant in the discovery cohorts and had the same direction of effect in both the discovery and validation analyses^25^. Race-stratified and multi-population meta-analyses were performed across the cohorts (Supplementary Data 1–3). Bonferroni *p*-values for the meta-analyses were based on the number of statistically significant phecodes in the discovery cohorts that were available and in the same direction in the validation cohorts (non-Hispanic Black: 2.55 × 10⁻⁴; non-Hispanic White: 1.33 × 10⁻⁴, multi-population 1.28 × 10⁻⁴). Secondary analyses, adjusting for only age, were also performed (Supplementary Data 4).

Using previously published methods in the R PheWAS package, phecodes were classified into 16 disease groups based largely off of organ systems and/or biologic processes^17,24^. A binomial test was used to test for directional relationships of significant associations across all tests, as well as to test for directional relationships within disease categories within stratified meta-analyses and multi-population meta-analysis. A Bonferroni correction was used to determine significance in tests within disease categories.

### Reporting summary

Further information on research design is available in the Nature Portfolio Reporting Summary linked to this article.

## Results

### Study Populations

We identified 52,295 females for discovery analyses in the SD database that had complete covariate (age and BMI) information to be included in analyses (9022 cases, 43,273 controls, Table 1). In the GHS validation cohort, there were 26,918 (10,232 cases, 16,686 controls) females with complete covariate information (Fig. 1A). Non-Hispanic Black individuals, classified using EHR-reported race and ethnicity, made up 19.88% and 3.51% of the discovery and validation cohorts. Average age at diagnosis was lower in non-Hispanic Black cases (SD: 39.2 years, GHS: 45.0 years) relative to non-Hispanic White cases (SD: 44.7 years, GHS: 52.7 years) in both cohorts. Average BMI in cases was higher relative to controls in both races (SD non-Hispanic Black and White individuals: 33.2 and 28.9 kg/m² in fibroid cases and 31.6 and 27.8 kg/m² in controls, GHS non-Hispanic Black and White individuals: 32.3 and 30.8 kg/m² in fibroid cases and 31.8 and 29.9 kg/m² in controls). In the discovery cohort, non-Hispanic Black individuals had a higher proportion of individuals with hypertension and Type 2 diabetes (5% and 11% in cases, 3% and 6% in controls) relative to non-Hispanic White individuals (2% and 6% in cases, 2% and 4% in controls). There was a higher burden of diabetes and hypertension in both cases and controls in GHS cohort compared to the cohort from the SD, likely due to the generally older and higher burden of obesity of the GHS patients (Table 1)^26^.

**Table 1:** Demographics of study populations

		Non-Hispanic Black Females				Non-Hispanic White Females			
Study	Characteristic	With fibroids		Without Fibroids		With fibroids		Without Fibroid	
SD***		*N* = 3599	(4%)*	*N* = 12,584	(16%)	*N* = 7307	(9%)	*N* = 57,544	(71%)
	Age (yrs) mean ± SD	39.2 ± 11.1		31.4 ± 13.9		44.7 ± 11.6		37.1 ± 15.7	
	<25	317	(9%)**	5153	(41%)	189	(4%)	12550	(25%)
	25–29	410	(11%)	2329	(19%)	501	(6%)	10482	(15%)
	30–34	544	(15%)	1518	12%)	778	(11%)	9216	(16%)
	35–39	620	(17%)	959	(8%)	1024	(14%)	6388	(11%)
	≥40	1708	(47%)	2625	(21%)	4815	(66%)	18,908	(33%)
	BMI (kg/m^2^) mean ± SD	33.2 ± 8.4		31.6 ± 9.2		28.9 ± 7.5		27.8 ± 7.6	
	Underweight	22	(1%)	159	(1%)	85	(1%)	1095	(2%)
	Normal	425	(12%)	1668	(13%)	2081	(29%)	14,467	(25%)
	Overweight	729	(20%)	1860	(15%)	1679	(23%)	9468	(17%)
	Obese	1791	(50%)	3743	(30%)	2210	(30%)	10,813	(19%)
	Missing	632	(18%)	5154	(41%)	1252	(17%)	21,701	(38%)
	Diabetes	164	(5%)	392	(3%)	176	(2%)	1158	(2%)
	Hypertension	380	(11%)	783	(6%)	463	(6%)	2261	(4%)
GHS****		*N* = 728	(2.6%)*	*N* = 242	(0.9%)	*N* = 9,688	(35%)	*N* = 17,056	(61.5%)
	Age (yrs) mean ± SD	45.0 ± 10.8		59.9 ± 10.4		52.7 ± 11.9		65.9 ± 12.9	
	<25	16	(2%)	0		56	(0.6%)	0	
	25–29	51	(7%)	0		174	(2%)	0	
	30–34	86	(12%)	0		439	(5%)	0	
	35–39	99	(14%)	0		777	(8%)	0	
	≥40	476	(65%)	242	(100%)	8242	(85%)	17,056	(100%)
	BMI (kg/m^2^) mean ± SD	32.3 ± 7.6		31.8 ± 8.2		30.8 ± 7.4		29.9 ± 7.8	
	Underweight	5	(0.7%)	2	(0.8%)	59	(0.6%)	306	(2%)
	Normal	96	(13%)	45	(19%)	2282	(24%)	4472	(26%)
	Overweight	213	(29%)	61	(25%)	2609	(27%)	4728	(28%)
	Obese	398	(55%)	125	(52%)	4570	(47%)	6947	(41%)
	Missing	16	(2%)	9	(4%)	168	(2%)	603	(4%)
	Diabetes	117	(16%)	88	(36%)	1463	(15%)	3675	(22%)
	Hypertension	274	(38%)	162	(67%)	3734	(39%)	9234	(54%)

^*^Percentages are out of the total number of individuals in the respective electronic health record population.

**Percentages are out of the total number of individuals in that column.

***Synthetic Derivative (Discovery) at Vanderbilt University Medical Center.

****Geisinger Health Systems (Validation).

### SD database discovery PheWAS

In discovery analyses using the SD, a total of 1678 and 1743 traits were tested for their associations with uterine fibroids in non-Hispanic Black and White individuals, respectively (Supplementary Data 1, 2). Two hundred and eight associations in non-Hispanic Black and 425 in non-Hispanic White individuals were statistically significant after correction for multiple testing. One hundred and ninety phecodes were statistically significant in both races in the discovery dataset (Supplementary Data 1, 2). Seventeen were statistically significant only in non-Hispanic Black females but not non-Hispanic White females, whereas 234 were statistically significant in non-Hispanic White females but not non-Hispanic Black females. One association (285-other anemias) was statistically significant in both non-Hispanic Black and non-Hispanic White individuals but had different directions of effect (positive association in non-Hispanic Black individuals and negative association in non-Hispanic White individuals). In the multi-population analysis, in which 1671 traits were tested for their association with fibroids. There were 482 statistically significant associations (Supplementary Data 3).

### GHS database validation PheWAS

In validation analyses, 197 of phecodes statistically significant in the non-Hispanic Black discovery cohort were available in the non-Hispanic Black validation cohort. One-hundred and sixty-nine of these codes had the same direction of effect as in the discovery cohort (Supplementary Data 1), though only nine of these codes were also statistically significant in the validation PheWAS. In non-Hispanic White females, 377 of the 420 available phecodes that were statistically significant in the discovery cohort had the same direction of effect in those from the GHS database (Supplementary Data 2). Two hundred and eleven phecodes with the same direction of effect were also statistically significant in the non-Hispanic White cohort from the GHS database. Of the 437 available phecodes that were statistically significant in the discovery multi-population analysis, 392 replicated as their direction remained consistent across EHR databases (Supplementary Data 3). Two hundred and eleven of the replicated codes in the multi-population cohort were also statistically significant in this validation PheWAS.

### Multi-population SD and GHS meta-analyses

In the multi-population meta-analysis, almost all of the 392 (389, 99.23%) phecode associations that were meta-analyzed across the discovery and validation cohorts were statistically significant at a Bonferroni significance level (Fig. 1A, Tables 2, 3). Three phecodes, atherosclerosis of native arteries of the extremities with ulceration or gangrene (*p*-value = 3.03 x 10^-4^), fracture of the foot (*p*-value = 4.66 x 10^-4^), and open wounds of head; neck; and trunk (*p*-value = 6.25 x 10^-4^), had suggestive significance but failed to reach statistical significance at the Bonferroni level. Demonstrating the strength and performance of our algorithm for defining cases and controls, the association with the largest odds ratio (OR) benign neoplasm of the uterus (OR_multi_ = 4625.78, 95% confidence interval [CI] = 3507.46–6100.66, *p*-value = 3.87 ×10^-778^). These associations were observed within individual races, in multi-population meta-analyses, and across the discovery and validation cohorts (Tables 2, 4). Additionally, several known fibroid risk factors were also associated with fibroid status, including disorders of menstruation and other abnormal bleeding from female genital tract (OR_multi_ = 6.45, 95% CI = 6.13–6.78, *p*-value = 5.60 × 10⁻¹¹³⁰), endometriosis (OR_multi_ = 7.89, 95% CI = 7.02–8.88, *p*-value = 2.59 × 10⁻²⁵⁸), diagnoses of overweight, obesity, and other hyperalimentation (OR_multi_ = 1.52, 95% CI = 1.45–1.60, *p*-value = 2.21 × 10^−58^), disorders of lipid metabolism (OR_multi_ = 1.47, 95% CI = 1.40–1.54, *p*-value = 4.61 × 10⁻⁵⁹), and vitamin D deficiency (OR_multi_ = 1.43, 95% CI = 1.35–1.51, *p*-value = 4.49 × 10⁻³⁶). In addition to validating known risk factors for fibroids (Table 2), we also discovered several associations within the multi-population meta-analyses that, to the best of our knowledge, have not been previously linked to fibroids (Table 3). Many of these associations, which we herein refer to as novel, were also statistically significant within the race-stratified meta-analyses.

**Table 2:** Multi-population associations of known symptoms and diagnoses with odds of uterine fibroids diagnosis

Phecode	Description	Phecode cases	Phecode controls	OR (95% CI)	*P* value	I²	I² *p* value
**Circulatory system**							
458.9	Hypotension NOS	1636	70,610	0.53 (0.46, 0.62)	4.74E−18	78.10	3.38E−03
**Dermatologic**							
701	Other hypertrophic & atrophic conditions of skin	2976	68,771	2.02 (1.86, 2.19)	1.13E−61	0.00	6.70E−01
**Endocrine/metabolic**							
241	Nontoxic nodular goiter	2920	59,857	1.81 (1.66, 1.97)	3.82E−41	0.00	7.00E−01
241.2	Nontoxic multinodular goiter	1671	59,857	1.98 (1.77, 2.21)	5.47E−34	0.00	9.00E−01
261.4	Vitamin D deficiency	8608	60,154	1.43 (1.35, 1.51)	4.49E−36	93.70	2.73E−10
272	Disorders of lipoid metabolism	19,190	53,592	1.47 (1.40, 1.54)	4.61E−59	65.80	3.00E−02
272.1	Hyperlipidemia	19,057	53,592	1.47 (1.40, 1.54)	1.09E−58	70.00	2.00E−02
272.13	Mixed hyperlipidemia	5235	53,592	1.64 (1.52, 1.76)	2.58E−41	94.70	2.73E−12
278	Overweight, obesity & other hyperalimentation	15,899	55,481	1.52 (1.45, 1.60)	2.21E−58	93.30	1.13E−09
**Genitourinary/Reproductive**							
599	Other symptoms/disorders or the urinary system	12,959	54,074	1.91 (1.82, 2.00)	9.95E−162	59.10	6.00E−02
599.3	Dysuria	6164	54,074	1.97 (1.85, 2.10)	3.74E−97	35.50	2.00E−01
599.4	Urinary incontinence	3175	54,074	2.12 (1.95, 2.31)	7.54E−67	59.40	6.00E−02
599.5	Frequency of urination and polyuria	3871	54,074	1.98 (1.83, 2.14)	3.67E−66	0.00	5.30E−01
614	Inflammatory diseases of female pelvic organs	7159	60,787	2.40 (2.27, 2.55)	5.03E−190	86.20	7.43E−05
614.1	Pelvic peritoneal adhesions, female (postoperative) (postinfection)	416	60,787	3.43 (2.79, 4.21)	1.52E−31	75.60	6.42E−03
614.3	Pelvic inflammatory disease	684	60,787	3.57 (3.03, 4.21)	3.74E−52	69.90	1.87E−02
614.32	Chronic inflammatory pelvic disease	232	60,787	5.92 (4.50, 7.79)	4.17E−37	88.90	6.15E−06
614.5	Inflammatory disease of cervix, vagina, and vulva	5733	60,787	2.09 (1.96, 2.23)	4.14E−109	0.00	6.70E−01
614.51	Cervicitis and endocervicitis	534	60,787	2.51 (2.08, 3.04)	3.84E−21	0.00	9.30E−01
614.52	Vaginitis and vulvovaginitis	4314	60,787	2.06 (1.92, 2.22)	4.24E−82	0.00	4.60E−01
615	Endometriosis	1417	60,787	7.89 (7.02, 8.88)	2.59E−258	90.10	1.27E−06
619	Noninflammatory female genital disorders	4959	63,970	3.72 (3.48, 3.97)	1.37E−337	94.80	1.43E−12
619.2	Disorders of uterus, not elsewhere classified	1452	63,970	4.96 (4.44, 5.55)	1.44E−175	83.40	4.34E−04
619.3	Noninflammatory disorders of cervix	389	63,970	3.13 (2.51, 3.89)	2.47E−24	0.00	3.90E−01
619.4	Noninflammaotry disorders of vagina	960	63,970	2.55 (2.20, 2.96)	9.15E−36	0.00	5.70E−01
621	Endometrial hyperplasia	628	71,408	6.94 (5.81, 8.29)	1.76E−101	87.40	2.79E−05
625	Pain & other symptoms associated with female genital organs	7203	57,548	3.02 (2.85, 3.20)	9.09E−311	44.80	1.40E−01
626	Disorders of menstruation & other abnormal bleeding from female genital tract	19,566	36,491	6.45 (6.13, 6.78)	5.60E−1130	98.10	8.11E−35
626.1	Irregular menstrual cycle/bleeding	16824	36,491	6.64 (6.31, 7.00)	2.24E−1111	97.90	2.36E−31
626.11	Absent or infrequent menstruation	2992	36,491	2.61 (2.37, 2.88)	2.91E−82	82.10	7.78E−04
626.12	Excessive or frequent menstruation	7184	36,491	12.16 (11.40, 12.96)	1.87E−1262	97.90	1.14E−30
626.13	Irregular menstrual cycle	5635	36,491	5.29 (4.93, 5.67)	3.94E−475	95.10	4.26E−13
626.14	Irregular menstrual bleeding	798	36,491	8.85 (7.56, 10.36)	6.19E−163	75.30	6.96E−03
626.2	Dysmenorrhea	2362	36,491	10.16 (9.19, 11.23)	2.17E−448	93.10	1.92E−09
626.8	Infertility, female	1475	36,491	3.26 (2.88, 3.68)	4.03E−79	97.10	3.44E−22
628	Ovarian cyst	5652	36,491	6.65 (6.21, 7.12)	6.52E−636	96.70	2.23E−19
**Hematopoietic**							
280	Iron deficiency anemias	5212	52,531	1.75 (1.63, 1.87)	2.35E−59	87.20	3.23E−05
**Neoplasms**							
182	Malignant neoplasm of uterus	420	55,080	247.70 (182.98, 335.30)	9.15E−279	94.10	5.97E−11
218	Benign neoplasm of uterus	9025	56,171	4625.78 (3507.46, 6100.66)	3.87E−778	45.00	1.40E−01
**Symptoms**							
798	Malaise and fatigue	17,062	48,658	1.42 (1.36, 1.49)	2.02E−56	75.20	7.00E−03

^*^OR odds ratio, *CI* confidence interval.

**Table 3:** Select multi-population associations of novel symptoms and diagnoses with odds of developing uterine fibroids

Phecode	Description	Phecode cases	Phecode controls	OR (95% CI)	*P* value	I²	I² *p* value
**Circulatory**							
411	Ischemic Heart Disease	5420	68,757	0.66 (0.61, 0.71)	6.84E−25	88.60	8.15E−06
415	Pulmonary heart disease	2292	71,802	0.66 (0.59, 0.74)	8.36E−13	51.90	1.00E−01
427.9	Palpitations	5482	56,327	1.55 (1.45, 1.65)	9.66E−39	0.00	7.90E−01
428	Congestive heart failure; nonhypertensive	3713	69,945	0.48 (0.43, 0.53)	2.38E−43	65.30	3.00E−02
443	Peripheral vascular disease	1655	69,804	0.70 (0.62, 0.80)	2.61E−07	88.30	1.13E−05
455	Hemorrhoids	2774	62,176	1.68 (1.54, 1.84)	6.73E−30	0.00	6.50E−01
**Dermatological**							
701	Other hypertrophic & atrophic conditions of the skin	2976	68,771	2.02 (1.86, 2.19)	1.13E−61	0.00	6.70E−01
702	Degenerative skin conditions and other dermatoses	4095	64,011	2.25 (2.09, 2.43)	6.29E-95	0.00	6.96E−01
706	Diseases of sebaceous glands	4252	67,312	1.80 (1.67, 1.94)	3.04E−55	0.00	4.40E−01
**Digestive**							
564.1	Irritable Bowel Syndrome	2441	58,131	1.73 (1.57, 1.91)	7.44E−29	35.90	1.97E−01
579	Other symptoms involving abdomen & pelvis	3348	61,609	2.26 (2.08, 2.45)	2.56E-87	89.40	3.32E−06
**Endocrine-Metabolic**							
250	Diabetes mellitus	9459	64,056	0.84 (0.79, 0.89)	8.94E−09	77.10	4.48E−03
261	Vitamin deficiency	9545	60,154	1.38 (1.31, 1.46)	1.74E−32	92.70	6.86E−09
276	Disorders of fluid, electrolyte, and acid-base balance	11,700	57,868	0.68 (0.65, 0.72)	9.67E−41	92.40	1.24E−08
**Genitourinary**							
585	Renal failure	6425	64,175	0.57 (0.53, 0.61)	9.50E−46	87.60	2.34E−05
610	Benign mammary dysplasias	2790	58,855	3.00 (2.76, 3.27)	9.96E−145	47.70	1.30E−01
611	Abnormal findings on mammogram or breast exam	8754	58,855	2.27 (2.15, 2.40)	3.81E−197	58.20	7.00E−02
612	Breast conditions, congenital or hormone related	894	58,855	2.20 (1.89, 2.56)	1.15E−24	27.10	2.49E−01
613	Other nonmalignant breast conditions	3079	70,329	2.06 (1.90, 2.24)	1.22E−69	77.40	4.04E−03
618	Genital prolapse	1687	74,962	3.56 (3.20, 3.95)	1.54E−120	0.00	5.50E−01
622	Polyp of female genital organs	1634	71,408	7.88 (7.04, 8.82)	6.19E−281	97.80	1.40E−29
623	Hypertrophy of female genital organs	258	71,408	24.21 (16.82, 34.85)	6.21E−66	0.00	5.70E−01
627	Menopausal and postmenopausal disorders	11,591	36,491	7.34 (6.93, 7.78)	1.89E−1002	96.40	7.00E−18
**Hematopoietic**							
284	Aplastic anemia	815	52,531	0.48 (0.39, 0.60)	2.65E−11	0.00	4.40E−01
285.2	Anemia of chronic disease	2227	52,531	0.66 (0.58, 0.74)	5.28E−12	0.00	5.40E−01
**Infectious**							
38	Septicemia	2636	68,519	0.46 (0.41, 0.52)	1.17E−36	1.90	3.80E−01
78	Viral warts & HPV	1783	60,638	1.61 (1.44, 1.80)	5.42E−17	34.30	2.10E−01
**Mental**							
300	Anxiety, phobic and dissociative disorders	13,985	45,201	1.29 (1.23, 1.35)	2.61E−24	73.40	1.02E−02
304	Adjustment reaction	4522	45,201	1.53 (1.42, 1.65)	1.14E−29	49.90	1.10E−01
**Musculoskeletal**							
726	Peripheral enthesopathies and allied syndromes	8129	57,726	2.02 (1.91, 2.13)	1.08E−135	75.00	7.40E−03
745	Pain in joint	21549	46,291	1.62 (1.56, 1.69)	1.38E−118	0.00	5.80E−01
**Neoplasm**							
174	Breast cancer	2040	63,626	1.45 (1.30, 1.62)	1.03E−11	78.40	3.03E−03
180	Cervical cancer and dysplasia	2705	50,918	7.92 (7.10, 8.83)	2.96E−303	0.00	6.10E−01
184	Cancer of other female genital organs	638	66,730	3.70 (3.13, 4.39)	6.01E−52	0.00	5.40E−01
208	Benign neoplasm of colon	3387	69,891	1.86 (1.72, 2.02)	8.68E−52	75.90	6.02E−03
216	Benign neoplasm of skin	4958	66,387	2.32 (2.17, 2.48)	6.23E−132	32.40	2.20E−01
220	Benign neoplasm of ovary	397	53,454	25.32 (20.42, 31.39)	7.22E−191	79.50	2.16E−03
**Neurologic**							
327	Sleep disorders	7195	63,182	1.57 (1.48, 1.67)	1.20E−52	66.60	3.00E−02
340	Migraine	5417	59,906	1.54 (1.45, 1.65)	2.30E−38	24.30	2.70E−01
**Pregnancy Complications**							
636	Early/threatened labor; hemorrhage early pregnancy	6159	64,400	0.73 (0.68, 0.80)	1.01E−13	83.20	4.70E−04
654.1	Abnormality of organs & soft tissues of pelvis complicating pregnancy, childbirth, or puerperium	2421	68,369	2.02 (1.82, 2.24)	2.21E−39	0.00	8.40E−01
655	Known or suspected fetal abnormality affecting management of mother	8191	64,929	0.56 (0.52, 0.61)	5.81E−47	80.70	1.40E−03
**Respiratory**							
464	Acute sinusitis	11776	46,402	2.18 (2.07, 2.29)	5.55E−192	66.60	3.00E−02
475	Chronic sinusitis	3609	52,265	1.94 (1.79, 2.10)	1.14E−61	18.00	3.00E−01
476	Allergic rhinitis	10126	52,265	1.95 (1.85, 2.05)	3.52E−144	31.30	2.20E−01
483	Acute bronchitis and bronchiolitis	5672	58,872	1.65 (1.54, 1.76)	2.05E−49	0.00	5.40E−01
507	Pleurisy; pleural effusion	2725	65,650	0.44 (0.39, 0.50)	1.32E−41	82.00	8.30E−04
508	Pulmonary collapse; interstitial & compensatory emphysema	3053	65,650	0.50 (0.45, 0.55)	9.25E−37	65.50	3.00E−02
509	Respiratory failure, insufficiency, arrest	3172	65,650	0.35 (0.32, 0.40)	2.03E−66	53.70	9.00E−02
**Symptoms**							
760	Back pain	15991	52,603	1.43 (1.37, 1.50)	2.19E−58	0.00	5.60E−01
770	Myalgia and myositis unspecified	5313	67,928	1.46 (1.37, 1.56)	1.80E−29	62.10	4.77E−02
785	Abdominal pain	26685	39,418	1.23 (1.18, 1.28)	7.36E−25	76.70	4.92E−03
792	Abnormal Papanicolaou smear & cervical HPV	5243	50,918	9.19 (8.44, 10.01)	3.68E−567	58.30	6.59E−02

^*^OR odds ratio, *CI* confidence interval.

**Table 4:** Select associations of symptoms and diagnoses with increased and decreased odds of developing uterine fibroids within race

Phecode	Description	Meta-Analysis Non-Hispanic Black Individuals						Meta-Analysis Non-Hispanic White Individuals					
		Phecode cases	Phecode controls	OR (95% CI)	*P* value	I²	I² *p* value	Phecode cases	Phecode controls	OR (95% CI)	*P* value	I²	I² *p* value
**Genitourinary**													
599.3	Dysuria	794	7692	1.77 (1.50, 2.09)	8.99E−12	56.4	0.13	5370	46,382	2.01 (1.88, 2.16)	1.86E-87	0	0.55
614	Inflammatory diseases of female pelvic organs	2209	6733	2.34 (2.09, 2.62)	1.18E−48	0	0.69	4950	54,054	2.43 (2.27, 2.60)	2.30E−143	95.3	3.98E−06
614.3	Pelvic inflammatory disease	235	6733	3.25 (2.47, 4.28)	3.25E−17	0	0.37	449	54,054	3.77 (3.07, 4.63)	8.98E−37	88.2	3.64E−03
615	Endometriosis	216	6733	9.51 (6.91, 13.08)	1.30E−43	0	0.49	1201	54,054	7.66 (6.75, 8.70)	5.84E−217	96.5	1.11E−07
618	Genital prolapse	101	10,740	3.90 (2.54, 5.99)	5.14E−10	0	0.98	1586	64,222	3.53 (3.17, 3.94)	3.53E−112	2.6	0.17
622	Polyp of female genital organs	195	9922	12.86 (8.68, 19.0)	3.80E−37	42.4	0.19	1439	61,486	7.54 (6.70, 8.48)	4.18E−247	137.0	6.40E−30
625	Pain and other symptoms associated with female genital organs	1278	7217	2.75 (2.41, 3.14)	2.92E−50	0	0.41	5925	50,331	3.09 (2.90, 3.29)	5.41E−263	5.6	0.12
626	Disorders of menstruation and other abnormal bleeding from female genital tract	3476	4716	7.33 (6.50, 8.26)	1.50E−232	62.8	0.10	16090	31775	6.27 (5.93, 6.63)	7.48E-901	167.3	2.76E−35
628	Ovarian cyst	750	4716	7.19 (6.01, 8.58)	1.60E−104	0	0.45	4902	31775	6.56 (6.09, 7.06)	1.08E−533	98.9	5.01E−21
**Endocrine-Metabolic**													
261	Vitamin deficiency	966	8938	1.92 (1.66, 2.22)	2.15E−18	79.9	0.03	8579	51,216	1.31 (1.24, 1.39)	1.26E−20	92.6	2.36E−04
261.4	Vitamin D deficiency	871	8938	2.00 (1.71, 2.33)	7.74E−19	86.2	0.01	7737	51,216	1.36 (1.28, 1.44)	1.57E−23	94.7	1.31E−05
272	Disorders of lipid metabolism	1671	8681	1.38 (1.22, 1.57)	4.78E−07	0	0.49	17,519	44,911	1.49 (1.41, 1.56)	8.69E−54	86.0	7.56E−03
276	Disorders of fluid, electrolyte, & acid-base balance	1998	7481	0.74 (0.66, 0.83)	4.25E−07	12.4	0.29	9702	50,387	0.67 (0.63, 0.71)	1.36E−35	97.3	1.49E−09
278	Overweight	2772	7190	1.35 (1.21, 1.51)	2.35E−07	49	0.16	13127	48,291	1.57 (1.48, 1.66)	9.90E−54	97.3	1.04E−09
278.4	Abnormal weight gain	335	7190	2.13 (1.67, 2.71)	1.29E−09	0	0.34	1872	48,291	1.89 (1.70, 2.11)	7.52E−31	50.6	0.15
**Neoplasm**													
180	Cervical cancer and dysplasia	767	5969	7.27 (5.95, 8.87)	2.40E-84	0	0.57	1938	44,949	8.21 (7.21, 9.35)	3.57E−221	0	0.48
182	Malignant neoplasm of uterus	41	6830	247.62 (59.00, 1039.22)	5.02E−14	0	0.99	379	48,250	247.70 (181.69, 337.67)	1.94E−266	98.0	1.14E−12
218	Benign neoplasm of uterus	1896	6878	11,741.68 (3402.88, 40514.86)	9.39E−50	0	0.78	7129	49293	4404.58 (3315.61, 5851.21)	7.26E−731	67.7	0.08
220	Benign neoplasm of ovary	88	6689	20.23 (13.04, 31.38)	4.86E−41	81.5	0.02	309	46,765	27.17 (21.24, 34.76)	5.10E−152	87.4	4.91E−03
**Symptoms**													
760	Back pain	2208	7318	1.36 (1.23, 1.52)	1.42E−08	0.3	0.32	13,783	45,285	1.45 (1.38, 1.52)	1.36E−51	0	0.80
761	Cervicalgia	822	9256	1.85 (1.59, 2.16)	3.23E−15	0	0.49	5805	56,083	1.56 (1.47, 1.67)	4.62E−44	97.1	3.21E−09
764	Sciatica	173	9893	2.24 (1.61, 3.12)	1.97E−06	0	0.75	2071	58,128	1.68 (1.51, 1.86)	2.68E−22	0	0.85
798	Malaise and fatigue	2413	6642	1.33 (1.19, 1.48)	1.73E−07	61.8	0.11	14,649	42,016	1.44 (1.37, 1.51)	7.05E−51	86.9	5.82E−03
**Respiratory**													
464	Acute sinusitis	761	6565	2.35 (1.98, 2.78)	3.92E−23	65.8	0.09	11015	39,837	2.16 (2.05, 2.28)	7.91E−171	80.9	0.02
475	Chronic sinusitis	333	7606	2.21 (1.75, 2.79)	2.45E−11	0	0.97	3276	44,659	1.91 (1.76, 2.08)	3.04E−52	56.8	0.13
**Hematopoietic**													
280	Iron deficiency anemias	1017	5766	2.17 (1.88, 2.51)	2.44E−25	0	0.47	4195	46,765	1.65 (1.53, 1.78)	4.39E−36	91.8	4.62E−04
**Neurological**													
327	Sleep disorders	651	9305	1.86 (1.56, 2.21)	3.02E−12	0	0.87	6544	53,877	1.54 (1.45, 1.64)	6.82E−43	79.9	3.00E−02
327.32	Obstructive sleep apnea	471	9305	1.55 (1.26, 1.89)	2.66E−05	6.5	0.30	2614	53,877	1.44 (1.31, 1.58)	4.66E−14	95.5	2.17E−06
340	Migraine	565	7353	1.83 (1.52, 2.21)	2.76E−10	0	0.96	4852	52,553	1.51 (1.41, 1.62)	1.91E−30	0	0.55
**Dermatological**													
694	Dyschromia and Vitiligo							823	56704	1.87 (1.60, 2.19)	5.95E−15	23.9	0.25
701	Other hypertrophic & atrophic conditions of skin	339	9950	1.90 (1.51, 2.38)	4.73E−08	0	0.32	2637	58,821	2.04 (1.86, 2.23)	3.05E−53	0	0.63
704.1	Alopecia	154	9779	2.81 (2.00, 3.94)	2.84E−09	0	0.42	1002	59,398	1.58 (1.36, 1.83)	7.90E−10	59.0	0.12

^*^OR odds ratio, *CI* confidence interval.

**Fig. 2:**
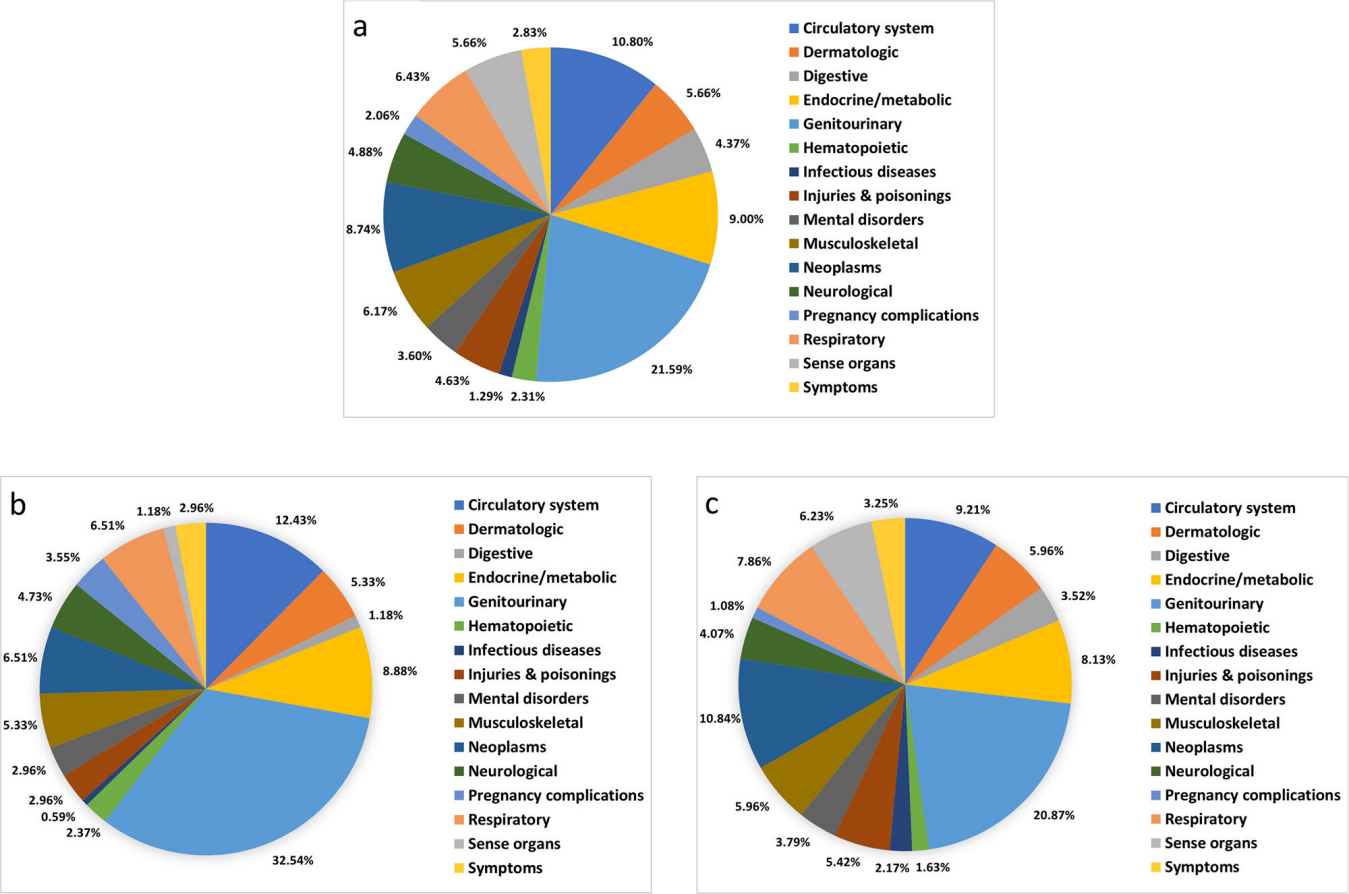
Breakdown of significant, replicated PheWAS outcomes by diagnosis group for meta-analyses. Pie charts illustrating the percentage of statistically significant, replicated associations within each of the 16 disease groups. The percentage of significant results in each category is displayed for the multi-population [N max=79,213] (**a**), non-Hispanic Black [N max = 11,342] (**b**), and non-Hispanic White [N max=67,871] (**c**) meta-analyses.

The most statistically significant associations within and across races and datasets were genitourinary diagnoses typically associated with fibroid symptoms (Fig. 2A, Tables 3, 4) including irregular menstrual cycle (OR_multi_ = 6.64, 95% CI = 6.31–7.00, *p*-value = 5.60 × 10⁻¹¹¹¹), excessive or frequent menstruation (OR_multi_ = 12.1, 95% CI = 11.40–12.96], *p*-value = 1.87 × 10⁻¹²⁶²), dysmenorrhea (OR_multi_ = 10.16, 95% CI = 9.19–11.23, *p*-value = 2.17 × 10⁻⁴⁴⁸), pain and other symptoms of female genital organs (OR_multi_= 3.02, 95% CI = 2.85–3.20, *p*-value = 9.09 × 10⁻³¹¹), and malaise and fatigue (OR_multi_ = 1.42, 95% CI = 1.36–1.49, *p*-value = 2.02 × 10⁻⁵⁶). An array of other gynecological or reproductive diseases (Tables 2, 3) were also associated with increase odds of fibroids including endometriosis (OR_multi_ = 7.89, 95% CI = 7.02–8.88, *p*-value = 2.59 × 10⁻²⁵⁸), inflammatory diseases of female pelvic organs (OR_multi_ = 2.40, 95% CI = 2.27–2.55, *p*-value = 5.03 × 10⁻¹⁹⁰), noninflammatory disorders of female genitals (OR_multi_ = 3.72, 95% CI = 3.48–3.97, *p*-value = 1.37 × 10⁻³³⁷), endometrial hyperplasia (OR_multi_ = 6.94, 95% CI = 5.81–8.29, *p*-value = 1.76 × 10⁻¹⁰¹), and ovarian cysts (OR_multi_= 6.65, 95% CI = 6.21–7.12, *p*-value = 6.52 ×10⁻⁶³⁶).

Hypotension not otherwise specified (OR_multi_ = 0.53, 95% CI = 0.46–0.62, *p*-value = 4.74 × 10⁻¹⁸), a known risk factor for fibroids, was associated with a reduced risk of fibroids. Multiple other, novel circulatory system diseases and symptoms were associated with fibroids. Ischemic heart disease (OR_multi_ = 0.66, 95% CI = 0.61–0.71, *p*-value = 6.84 × 10⁻²⁵), pulmonary heart disease (OR_multi_ = 0.66, 95% CI = 0.59–0.74, *p*-value = 8.36 × 10⁻¹³), non-hypertensive congestive heart failure (OR_multi_ = 0.48, 95% CI = 0.43–0.53, *p*-value = 2.38 × 10⁻⁴³), and peripheral vascular disease (OR_multi_ = 0.70, 95% CI = 0.62–0.80, *p*-value = 2.61 × 10⁻⁷) diagnoses are also associated with reduced risk of fibroid diagnosis. Hemorrhoids (OR_multi_ = 1.68, 95% CI = 1.54–1.84, *p*-value = 6.73 ×10⁻³⁰) and palpitations (OR_multi_ = 1.55, 95% CI = 1.45–1.65, *p*-value = 9.66 × 10⁻³⁹) are the only two circulatory diagnoses that show increased odds (Tables 2, 3).

Uterine fibroids were also associated with neoplastic growths in both genitourinary and neoplasia diagnosis categories. The association with the highest odds of fibroids, outside of benign neoplasms of the uterus under which the code for uterine fibroids fall, was malignant neoplasm of the uterus (OR_multi_ = 247.70, 95% CI = 182.98–335.30, *p*-value = 9.15 × 10⁻²⁷⁹). Polyps of female genital organs (OR_multi_ = 7.88, 95% CI = 7.04–8.82, *p*-value = 6.19 × 10⁻²⁸¹) was associated with increased odds of fibroids. Consistent with previous evidence of links between keloids and fibroids, we find a positive association between other hypertrophic skin conditions (phecode 701 related to scars and keloids) and uterine fibroids (OR_multi_ = 2.02, 95% CI = 1.86–2.19, *p*-value = 1.13 × 10⁻⁶¹). Other benign growths such as benign neoplasms of the ovary (OR_multi_ = 25.32, 95% CI = 20.42–31.39, *p*-value = 7.22 × 10⁻¹⁹¹) and mammary dysplasia (OR_multi_ = 3.00, 95% CI = 2.76–3.27, *p*-value = 9.96 × 10⁻¹⁴⁵) are positively associated with uterine fibroids.

Respiratory diagnoses were also significantly associated with fibroids. Most respiratory diagnoses showed increased odds of fibroids, including acute (OR_multi_ = 2.18, 95% CI = 2.07–2.29, *p*-value = 5.55 × 10⁻¹⁹²) and chronic sinusitis (OR_multi_ = 1.94, 95% CI = 1.79–2.10, *p*-value = 1.14 × 10⁻⁶¹), acute bronchitis and bronchiolitis (OR_multi_ = 1.65, 95% CI = 1.54–1.76, *p*-value = 2.05 × 10⁻⁴⁹), and allergic rhinitis (OR_multi_ = 1.95, 95% CI = 1.85–2.05, *p*-value = 3.52 × 10⁻¹⁴⁴). However, there were a few respiratory diagnoses that showed lower odds of fibroids including pleurisy, pulmonary collapse, and respiratory failure (ORs_multi_ = 0.35–0.50, 95% CIs = 0.32–0.55 *p*-values < 5.00 × 10⁻³⁶).

### Racially Stratified Meta-Analyses

All 169 phecodes that were meta-analyzed in the non-Hispanic Black cohort were significantly associated with uterine fibroids after adjustment for multiple testing (Fig. 1b). Of the 377 phecodes which were meta-analyzed in the non-Hispanic White cohort, 369 (~98%) were significantly associated with fibroids and only eight phecodes did not reach significance after adjusting for multiple testing (Fig. 1C). Known risk factors, including overweight/obesity, other hypertrophic and atrophic conditions of skin, and pelvic inflammatory disease, were significantly associated with fibroids in both groups (Table 4). Symptoms often associated with fibroids, such as dysuria, pain and other symptoms associated with female genital organs, ovarian cysts, and malaise and fatigue, were also associated with fibroid diagnosis in both groups. In general, the association between fibroid status and several known and novel factors was greater in non-Hispanic Black females relative to non-Hispanic White females (e.g., vitamin D deficiency OR_black_ = 2.00, 95% CI = 1.71–2.33, *p*-value = 7.74 x 10^⁻¹⁹^, OR_white_ = 1.36, 95% CI = 1.28–1.44, *p*-value = 1.57 x 10⁻²³; endometriosis OR_black_ = 9.51, 95% CI = 6.91–13.08, *p*-value = 1.30 × 10⁻⁴³, OR_white_ = 7.66, 95% CI = 6.75–8.70, *p*-value = 5.84 × 10⁻²¹⁷), though there were a few instances where the opposite was true (e.g., benign neoplasm of ovary OR_black_ = 20.23, 95% CI = 13.04–31.38, *p*-value = 4.86 × 10⁻⁴¹, OR_white_ = 27.17, 95% CI = 21.24–34.76, *p*-value = 5.10 × 10⁻¹⁵²). Several novel associations were observed in both non-Hispanic Black and White cohorts, including genitourinary diagnoses such as genital prolapse and polyps of female genital organs (Table 4, Supplementary Data 1, 2).

There was an overall enrichment for positive relationships in both meta-analyses, demonstrating a marked increase in comorbidities in individuals with uterine fibroids compared to those without fibroids (Table 5). Within non-Hispanic Black and White females and across EHR-defined races, genitourinary diagnoses represent the highest proportion of statistically significant replicated associations (Fig. 2b, c). Diagnoses in the circulatory, endocrine/metabolic, respiratory, neoplasm, and musculoskeletal groups followed, but exact rank varied by race. All diagnoses within the sense organ and musculoskeletal group were positively associated with fibroids, suggesting that fibroids and at least one musculoskeletal or sense organ diagnosis co-occur. Both within and across races, diagnoses in the genitourinary group tended to be positively associated with fibroids (Tables 4, 5), while circulatory diagnoses were associated with negatively correlated and tied to decreased odds of fibroids (Tables 4, 5). Diagnoses in the dermatologic and sense organ groups were only positively associated with fibroids in non-Hispanic White females (Table 5).

**Table 5:** Sign tests for directionality within and across disease groups

	Non-Hispanic Black Individuals Meta-analysis							Non-Hispanic White Individuals Meta-analysis				Meta-analysis									
Disease group	Positive, significant tests (*N*)	Significant tests (*N*)	Test ran (N)	*P* Value	Positive significant test (without subcodes) (*N*)	Significant tests (without subcodes) (*N*)	*P* Value	Positive, significant tests (*N*)	Significant tests (*N*)	Test ran (*N*)	*P* Value	Positive significant tests (without subcodes) (*N*)	Significant tests (without subcodes) (*N*)	*P* Value	Positive, significant tests (*N*)	Significant tests (*N*)	Test ran (*N*)	*P* Value	Positive significant tests (without subcodes) (N)	Significant tests (without subcodes) (N)	P Value
**Circulatory system**	2	21	21	**2.21E−04**	2	11	0.07	3	34	39	**7.66E−07**	3	18	7.54E−03	4	42	43	**5.65E−08**	4	23	2.60E−03
**Dermatologic**	7	9	9	0.18	5	6	0.22	20	22	22	**1.21E−04**	12	12	**4.88E−04**	20	22	22	**1.21E−04**	11	12	6.35E−03
**Digestive**	2	2	2	0.50	2	2	0.50	10	13	15	0.09	8	11	0.23	10	17	17	0.63	7	14	1.00
**Endocrine/ metabolic**	8	15	15	1.00	4	5	0.38	15	30	30	1.00	8	11	0.23	19	35	35	0.74	9	12	0.15
**Genitourinary**	47	55	55	**8.07E−08**	17	19	**7.29E−04**	73	77	77	**<2.2E−16**	22	23	**5.72E−06**	71	82	82	**6.81E−12**	20	23	4.88E−04
**Hemato-poietic**	4	4	4	0.13	2	2	0.50	2	6	6	0.69	1	4	0.63	3	9	9	0.51	1	4	0.63
**Infectious diseases**	0	1	1	1.00	0	1	1.00	3	8	8	0.73	2	5	1.00	3	5	5	1.00	2	3	1.00
**Injuries & poisonings**	2	5	5	1.00	2	4	1.00	4	20	20	0.01	4	13	0.27	5	18	20	9.63E−02	4	11	0.55
**Mental disorders**	0	5	5	0.06	0	2	0.50	7	14	14	1.00	5	10	1.000	8	14	14	0.79	5	9	1.00
**Musculo- skeletal**	9	9	9	**3.91E−03**	6	6	0.03	22	22	22	**4.77E−07**	9	9	**3.91E−03**	24	24	24	**1.19E−07**	10	10	1.95E−03
**Neoplasms**	7	11	11	0.55	5	7	0.45	28	40	40	0.02	17	22	0.02	21	34	34	0.23	13	18	9.63E−02
**Neurological**	5	8	8	0.73	4	6	0.69	12	15	15	0.04	4	6	0.69	13	19	19	0.17	4	7	1.00
**Pregnancy complications**	1	6	6	0.22	1	4	0.63	1	4	5	0.63	1	3	1.00	1	8	8	7.03E−02	1	7	7.03E−02
**Respiratory**	7	11	11	0.55	6	9	0.51	13	29	29	0.71	10	20	1.00	14	25	25	0.69	11	19	0.65
**Sense organs**	2	2	2	0.50	1	1	1.00	23	23	23	**2.38E−07**	10	10	**1.95E−03**	22	22	22	**4.77E−08**	11	11	9.77E−04
**Symptoms**	5	5	5	0.06	5	5	0.06	10	12	12	0.04	10	12	0.04	12	13	13	3.42E−03	11	12	6.35E−03
**Total**	108	169	169	3.72E−04	62	90	**4.38E−04**	246	369	377	**1.46E−10**	126	189	**2.17E−03**	250	389	392	**1.97E−08**	124	195	1.81E−04

## Discussion

Using a validated, multi-stage PheWAS, we found statistically significant associations between fibroid status and multiple disease categories, with the strongest risk factors being among genitourinary, musculoskeletal, and neoplasms. Importantly, known fibroid risk factors, such as inflammatory diseases of female pelvic organs, disorders of menstruation, and other abnormal bleeding from the female genital track, dysmenorrhea, hyperlipidemia, and vitamin D deficiency, were the most strongly associated diagnoses, highlighting the validity of our method. Outside these known risk factors, we also identified several novel diagnoses associated with uterine fibroids, including neoplasms and diagnoses linked to autoimmunity. In general, females with uterine fibroids had a significantly higher number of co-morbid diagnoses relative to control individuals. Across disease groups, diagnoses tended to be positively associated with fibroids within and across race groups, suggesting that fibroids are associated with increased comorbidities in many disease groups.

Genitourinary diagnoses, such as symptoms related to menstruation (frequent, irregular, excessive), dysmenorrhea, pain in female genital organs, disorders of the urinary system, and early menopause, were the strongest associations. These diagnoses are the most typical symptoms frequently reported by individuals with symptomatic fibroids, further highlighting the validity of our phenotyping algorithm and PheWAS approach^27–29^. Our results also confirm previously identified relationships between other genitourinary diagnoses and uterine fibroids. For example, there was strong relationship between leiomyoma and endometriosis. Previous studies that have identified a positive relationship between endometriosis and fibroids, as well as evidence of a common genetic basis between the two conditions^30–33^.

Known risk factors and related conditions, outside of genitourinary diagnoses, were also observed. For example, diagnoses related to BMI, an established risk factor for fibroids, were more common in individuals with fibroids regardless of race. These diagnoses, including obesity and disorders of lipid metabolism, are also established risk factors for leiomyoma^6,34–36^. Our models were adjusted for BMI, suggesting other pathways or nonlinear relationships with BMI between fibroids and these traits.

Vitamin D deficiency was significantly associated with fibroids. Case-control studies have found lower Vitamin D levels in females with uterine fibroids^10,37–39^. Lower levels of Vitamin D have also been observed in Black females compared to White females^40^. Our study found that non-Hispanic Black individuals with Vitamin D deficiency had higher odds of fibroid diagnosis relative to non-Hispanic White individuals. In vitro studies have identified a role of Vitamin D in reducing the expression of key genes related to extracellular matrix production in fibroid cells^41^. Diagnosis with atrophic skin conditions also increased odds of uterine fibroid diagnosis^42,43^.

Our study identified several novel associations. Briefly, diagnoses such as inflammatory pelvic disease, non-inflammatory pelvic disease, and benign mammary dysplasia, which were not previously well-documented as associated with fibroids, were associated with increased odds of fibroids. As with leiomyomas, many of these genitourinary diagnoses are related to estrogen or hormone dysregulation (endometrial hyperplasia, endometriosis, pelvic inflammatory disease, cystic mastopathy)^44–51^. These findings suggest a plausible etiologic mechanism shared across genitourinary disease: hormone dysregulation^52^.

Fibroids are generally considered as benign neoplasms^3,5,53,54^. However, our findings suggest a common underlying biology of fibroids and both benign and malignant neoplasms. The second largest association, after benign neoplasms of the uterus (the parent code for fibroids), was malignant neoplasm of the uterus. Cervical cancer, cervical intraepithelial dysplasia, abnormal Papanicolaou smear of cervix uterine, cervical and genital polyps, as well as benign neoplasms of the ovary and breast, were also positively associated with fibroids. If fibroids or polyps develop prior to genitourinary and reproductive malignancies, these conditions could be risk factors and may be useful in screening tests.

Diagnoses in the circulatory system category, such as ischemic heart disease, peripheral vascular disease, and congestive heart failure, were consistently associated with reduced odds of uterine fibroid diagnosis. Hypotension not otherwise specified was also negatively associated with fibroids, consistent with reports of high blood pressure and hypertension increasing risk of fibroids^11,55,56^. These results suggest an underlying mechanism linking biology of cardiovascular function and uterine fibroids. Estrogen, which has been associated with fibroid development, has known protective effects for cardiovascular disease^57^. Interestingly, obesity and metabolic disorders are typically associated with increased risk of vascular disease^58–60^. The links between leiomyoma, obesity, metabolic disease, and cardiovascular outcomes suggests more complex relationships between the physiology of these diagnoses that requires further research.

We did not observe a consistent pattern of increased or decreased odds of fibroids within other disease groups. However, when comparing associations across remaining disease groups, we observed increased odds with immune/inflammation pathways. For example, respiratory diagnoses such as acute bronchiolitis, chronic and acute sinusitis, and upper respiratory infections were associated with increased odds of fibroids. Similarly, other disease groups like symptoms (cervical radiculitis, thoracic neuritis/radiculitis, cervicalgia^61^), digestive (irritable bowel syndrome^62^), musculoskeletal (synovitis/tenosynovitis, pain and stiffness in joint^63^) and dermatological (dyschromia and vitiligo^64^, alopecia^65^) diagnoses, which are linked with immune and/or inflammation, are also associated with increased odds of fibroids in our study. Inflammatory processes and dysregulation have previously been suggested to be involved in the development of uterine fibroids^66^, endometrial disorders^67^, and cardiovascular disease via metabolic syndrome^68^.

In general, non-Hispanic Black and White females showed similar patterns in associations. The race-specific associations tended to have related diagnoses that were statistically significant in the multi-population meta-analysis or in non-Hispanic White females. For example, fluid overload is significant only in non-Hispanic Black females, however, the related diagnosis of “disorders of fluid, electrolyte, and acid base balance” is statistically significant in both races and meta-analysis. The disparity in associations between non-Hispanic Black and White females suggests both genetic and non-genetic differences. However, it is also possible that such a large disparity is a result of a lack of statistical power due to the smaller sample size of non-Hispanic Black cases relative to non-Hispanic White cases. Further research is needed to replicate these differences and uncover any racial disparities in diagnoses.

PheWAS provides a unique way to test for comorbidities and patterns of disease in a systematic fashion. This method is dependent on EHR diagnostic and billing codes, the entry of which do not always correspond to true disease presence. Reliance on these codes could lead to bias due to misclassification. However, validation in two different EHR databases, where clinical practice and coding is likely to vary, lessens the probability that significant results are due to bias or chance. Furthermore, fibroid cases and controls were identified using our previously published algorithm, which was previously shown to have high performance^22^. One component of this algorithm was the requirement that all individuals have pelvic imaging to be eligible for inclusion. This requirement eliminates the possibility of misclassification of cases and controls and the resulting bias introduced by asymptomatic disease. Controls also had to have an intact uterus. This further ensured that they were an accurate group to compare to those diagnosed with fibroids, unlike those without uteruses (i.e., those who had hysterectomies), who cannot have fibroids. A stringent significance threshold by Bonferroni correction was also adopted to further reduce the chance of false associations. The successful identification of the well-known risk factors indicates the validity of these methods to detect real relationships. However, the associations identified in this study do not implicate causality. A well-controlled longitudinal study may provide more insight in the causal direction between fibroids and other diseases.

We validated previously reported risk factors and identified novel diagnoses that have not been previously linked to uterine fibroids. In general, females with uterine fibroids bear a larger burden of comorbid traits across most disease-diagnosis groups. We detected novel significant associations of fibroids with malignant neoplasms in the uterus and cervix, as well as decreased negative associations with cardiovascular diagnoses and positive associations with inflammation-related diseases. This study provides the most detailed systematic research into fibroids and comorbidities to-date by leveraging large-scale EHR databases and PheWAS methodology and demonstrates a novel approach to identifying previously uncharacterized comorbidities of uterine fibroids.

## Supplementary information

Supplementary Information

Description of Additional Supplementary Files

Supplementary Data 1

Supplementary Data 2

Supplementary Data 3

Supplementary Data 4

Reporting Summary

## Data Availability

Individual-level data for this manuscript cannot be made readily available, but is readily available through IRB approval to either Vanderbilt University Medical Center or Geisinger Health System employees. Full results for all analyses (source data) are accessible and provided in Supplementary Data 1–4.

## Code availability

No new code was generated for this analysis. The phenotyping algorithm and phenome-wide association R code were previously published and are publicly available.
