# Supplementary material for: A phenome-wide association study of uterine fibroids reveals a marked burden of comorbidities
Source: Commun Med (Lond). 2025 May 15;5:174. doi: 10.1038/s43856-025-00884-w (PMC12081880; doi:10.1038/s43856-025-00884-w)
Supplement: Supplementary file 1 — Supplementary Information [file 43856_2025_884_MOESM1_ESM.pdf]

Supplementary Fig 1. Analyses and Results Flow Diagram

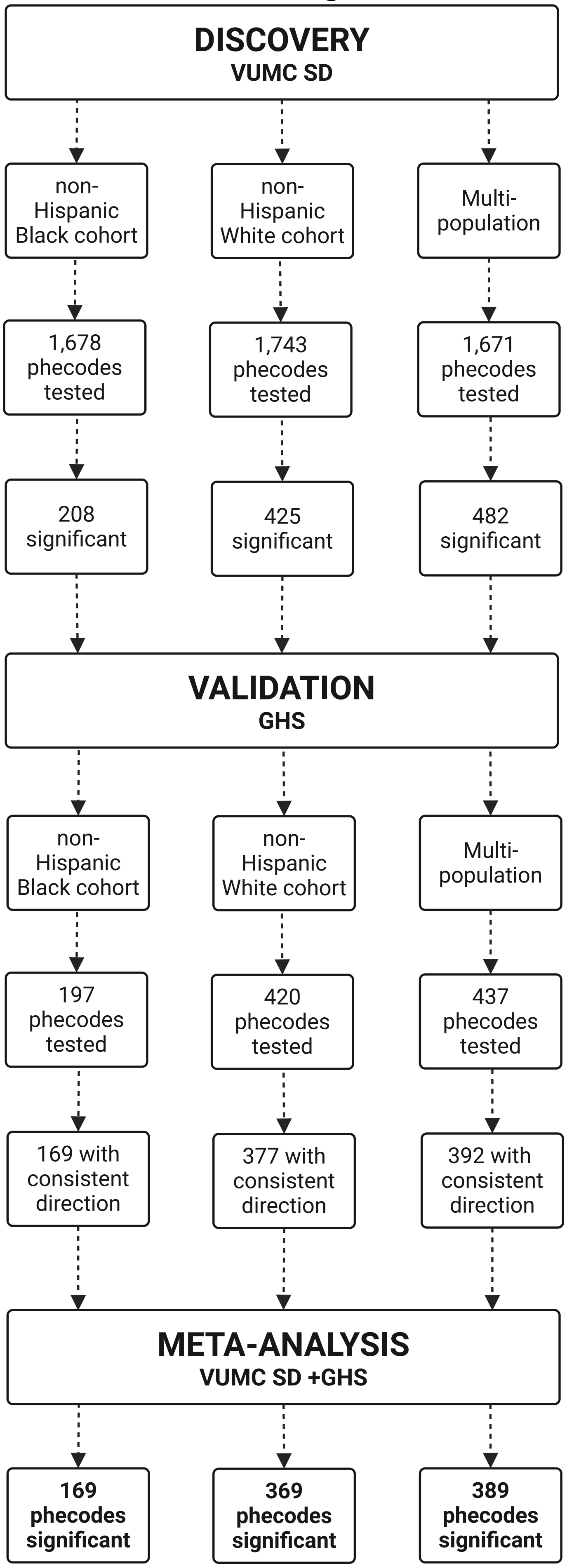

Supplemental Figure 1. legend: Flow diagram illustrating the analytical steps and the number of phecodes that were tested and/or significant in subsequent analysis.
