# Supplementary material for: A phenome-wide association study of uterine fibroids reveals a marked burden of comorbidities
Source: Commun Med (Lond). 2025 May 15;5:174. doi: 10.1038/s43856-025-00884-w (PMC12081880; doi:10.1038/s43856-025-00884-w)
Supplement: Supplementary file 2 — Description of Additional Supplementary Files [file 43856_2025_884_MOESM2_ESM.pdf]

Description of Additional Supplementary Files

File name: Supplementary Data 1.

File description: Full PheWAS from VUMC SD with values from GHS from replicated phecodes in Black females

File name: Supplementary Data 2.

File description: Full PheWAS from VUMC SD with values from GHS from replicated phecodes in White females

File name: Supplementary Data 3.

File description: Full Multi-Population PheWAS from VUMC SD with values from GHS from replicated phecodes

File name: Supplementary Data 4.

File description: PheWAS analyses adjusted for age only
