# Supplementary material for: A phenome-wide association study of uterine fibroids reveals a marked burden of comorbidities
Source: Commun Med (Lond). 2025 May 15;5:174. doi: 10.1038/s43856-025-00884-w (PMC12081880; doi:10.1038/s43856-025-00884-w)
Supplement: Supplementary file 7 — Reporting Summary [file 43856_2025_884_MOESM7_ESM.pdf]

nature portfolio

Corresponding author(s): Digna R. Velez Edwards

Last updated by author(s): Nov 1, 2024

Reporting Summary

Nature Portfolio wishes to improve the reproducibility of the work that we publish. This form provides structure for consistency and transparency in reporting. For further information on Nature Portfolio policies, see our Editorial Policies and the Editorial Policy Checklist.

Statistics

For all statistical analyses, confirm that the following items are present in the figure legend, table legend, main text, or Methods section.

n/a	Confirmed	
☐	☒	The exact sample size (*n*) for each experimental group/condition, given as a discrete number and unit of measurement
☐	☒	A statement on whether measurements were taken from distinct samples or whether the same sample was measured repeatedly
☐	☒	The statistical test(s) used AND whether they are one- or two-sided<br>*Only common tests should be described solely by name; describe more complex techniques in the Methods section.*
☐	☒	A description of all covariates tested
☐	☒	A description of any assumptions or corrections, such as tests of normality and adjustment for multiple comparisons
☐	☒	A full description of the statistical parameters including central tendency (e.g. means) or other basic estimates (e.g. regression coefficient) AND variation (e.g. standard deviation) or associated estimates of uncertainty (e.g. confidence intervals)
☐	☒	For null hypothesis testing, the test statistic (e.g. *F*, *t*, *r*) with confidence intervals, effect sizes, degrees of freedom and *P* value noted<br>*Give P values as exact values whenever suitable.*
☒	☐	For Bayesian analysis, information on the choice of priors and Markov chain Monte Carlo settings
☒	☐	For hierarchical and complex designs, identification of the appropriate level for tests and full reporting of outcomes
☐	☒	Estimates of effect sizes (e.g. Cohen's *d*, Pearson's *r*), indicating how they were calculated

Our web collection on statistics for biologists contains articles on many of the points above.

Software and code

Policy information about availability of computer code

Data collection	Vanderbilt University Medical Center's Synthetic Derivative and Geisinger Health System Database, two electronic health record databases, were used to obtain data. Non-Hispanic Black and White females over the age of 18 were eligible for inclusion in our study. Phenotyping of fibroid cases and controls was accomplished using a previous published algorithm. Code for the phenotyping within this manuscript is available with reasonable request from the corresponding author.
Data analysis	Analyses were performed using the PheWAS package (v 0.99.5-3) in R version 4.3.2. Inverse-variance weighted fixed-effects meta-analyses was performed using METAL software. Code for the analyses within this manuscript is available with reasonable request from the corresponding author.

For manuscripts utilizing custom algorithms or software that are central to the research but not yet described in published literature, software must be made available to editors and reviewers. We strongly encourage code deposition in a community repository (e.g. GitHub). See the Nature Portfolio guidelines for submitting code & software for further information.

Data

Policy information about availability of data

All manuscripts must include a data availability statement. This statement should provide the following information, where applicable:

- Accession codes, unique identifiers, or web links for publicly available datasets
- A description of any restrictions on data availability
- For clinical datasets or third party data, please ensure that the statement adheres to our policy

Individual-level data for this manuscript cannot be made readily available but is readily available through IRB approval to either Vanderbilt University Medical Center or Geisinger Health System employees. Full results for all analyses are accessible and provided in Supplementary Data 1-4.

Human research participants

Policy information about studies involving human research participants and Sex and Gender in Research.

Reporting on sex and gender	N/A. Non-human subject research
Population characteristics	N/A. Non-human subject research
Recruitment	N/A
Ethics oversight	Vanderbilt University Medical Center Institutional Review Board (IRB)

Note that full information on the approval of the study protocol must also be provided in the manuscript.

Field-specific reporting

Please select the one below that is the best fit for your research. If you are not sure, read the appropriate sections before making your selection.

☒ Life sciences ☐ Behavioural & social sciences ☐ Ecological, evolutionary & environmental sciences

For a reference copy of the document with all sections, see nature.com/documents/nr-reporting-summary-flat.pdf

Life sciences study design

All studies must disclose on these points even when the disclosure is negative.

Sample size	Only females were included in the study. Non-Hispanic Black and White females 18 years or older were eligible for inclusion, with race and ethnicity defined via self-reported or by providers. Cases and controls were identified using our previously published algorithm. Cases had at least one International Classification of Diseases, 9th Revision (ICD-9) or current procedure terminology (CPT) code for pelvic imaging and had at least one ICD-9 or CPT code indicating a fibroid diagnosis. Controls had at least two procedural codes for pelvic imaging without a fibroid diagnosis at time of last pelvic exam, as well as no history of hysterectomy, myomectomy, or uterine artery embolization. The discovery population have over 50,000 individuals and the validation population had over 26,000 individuals, which provided enough power for the analyses.
Data exclusions	In addition to the condition for inclusion listed above, only females with complete covariate information were included in analyses. For the main analyses, individuals needed to have age and body mass index information. In secondary analyses, individuals were only excluded if they did not have complete age information.
Replication	We utilized independent populations from two electronic health record database for discovery and validation of findings (Vanderbilt University Medical Center's Synthetic Derivative and Geisinger Health System Database) to verify our findings.
Randomization	N/A
Blinding	N/A

Reporting for specific materials, systems and methods

We require information from authors about some types of materials, experimental systems and methods used in many studies. Here, indicate whether each material, system or method listed is relevant to your study. If you are not sure if a list item applies to your research, read the appropriate section before selecting a response.

Materials & experimental systems

n/a	Involved in the study
☒	☐ Antibodies
☒	☐ Eukaryotic cell lines
☒	☐ Palaeontology and archaeology
☒	☐ Animals and other organisms
☒	☐ Clinical data
☒	☐ Dual use research of concern

Methods

n/a	Involved in the study
☒	☐ ChIP-seq
☒	☐ Flow cytometry
☒	☐ MRI-based neuroimaging
